# Culture Models to Investigate Mechanisms of Milk Production and Blood-Milk Barrier in Mammary Epithelial Cells: a Review and a Protocol

**DOI:** 10.1007/s10911-023-09536-y

**Published:** 2023-05-01

**Authors:** Ken Kobayashi

**Affiliations:** Laboratory of Cell and Tissue Biology, Research Faculty of Agriculture, Hokkaido University, North 9, West 9, Sapporo, 060-8589 Japan

**Keywords:** Mammary epithelial cell, Culture model, Milk production, Tight junction, Membrane proteins

## Abstract

Mammary epithelial cells (MECs) are the only cell type that produces milk during lactation. MECs also form less-permeable tight junctions (TJs) to prevent the leakage of milk and blood components through the paracellular pathway (blood-milk barrier). Multiple factors that include hormones, cytokines, nutrition, and temperature regulate milk production and TJ formation in MECs. Multiple intracellular signaling pathways that positively and negatively regulate milk production and TJ formation have been reported. However, their regulatory mechanisms have not been fully elucidated. In addition, unidentified components that regulate milk production in MECs likely exist in foods, for example plants. Culture models of functional MECs that recapitulate milk production and TJs are useful tools for their study. Such models enable the elimination of indirect effects via cells other than MECs and allows for more detailed experimental conditions. However, culture models of MECs with inappropriate functionality may result in unphysiological reactions that never occur in lactating mammary glands in vivo. Here, I briefly review the physiological functions of alveolar MECs during lactation in vivo and culture models of MECs that feature milk production and less-permeable TJs, together with a protocol for establishment of MEC culture with functional TJ barrier and milk production capability using cell culture inserts.

## Overview of Alveolar Luminal Mammary Epithelial Cell (MEC) Functions in Mammary Glands during Lactation

### Milk Production in Alveolar Luminal MECs during Lactation

Cells that secrete milk are luminal epithelial cells located in the mammary alveoli in the peripheral regions of mammary ducts [1, 2]. In addition, myoepithelial cells that contract and force milk to be ejected from the alveolar lumen are basally positioned in the mammary alveoli. In this review, unless otherwise mentioned, MEC refers to the luminal MECs that secrete milk. Alveolar MECs and myoepithelial cells gradually increase concurrently with alveolar structure development during pregnancy. After parturition, alveolar MECs initiate the production of caseins, whey proteins, lipids, and lactose as the major milk components. During lactation, alveolar MECs maintain milk production and myoepithelial cells eject milk for suckling offspring. However, alveolar MECs stop producing milk and are then lost after weaning, with the exception of luminal progenitor cells which undergo transcriptional changes in response to a full pregnancy, lactation and involution [3–5]. Alveolar MECs then reappear after pregnancy, and milk production is reintroduced into alveolar MECs. Thus, alveolar MECs are specialized cells that appear during pregnancy and produce milk only during lactation.

**Fig. 1:**
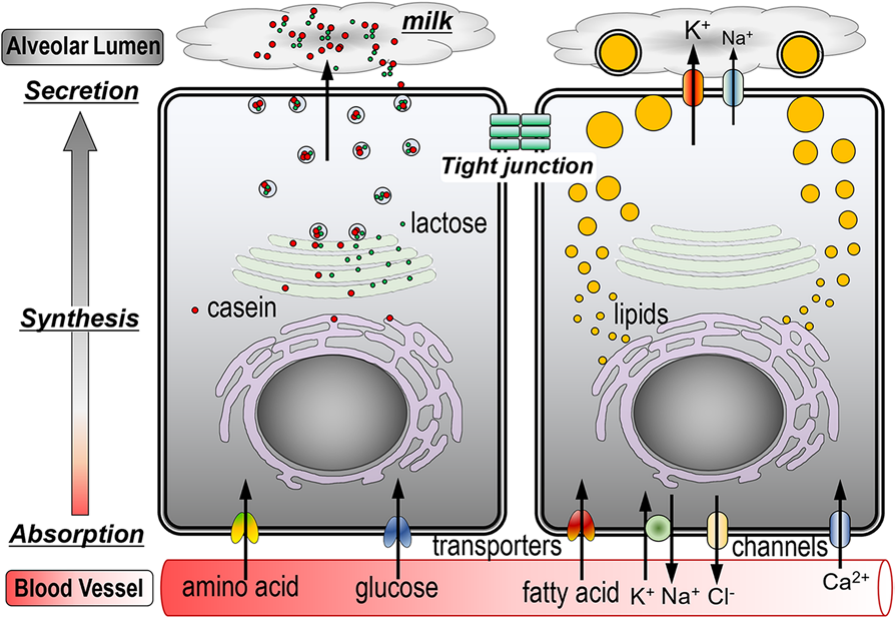
A schematic image of milk production pathways in MECs during lactation. MECs absorb nutrients and ions via specific channels and transporters on the basolateral membrane. MECs then synthesize milk proteins, lactose, and lipids in the endoplasmic reticulum and Golgi apparatus followed by secretion into the alveolar lumen via the apical membrane. TJs block leakage of the milk and blood components via the paracellular pathways between MECs

**Fig. 2:**
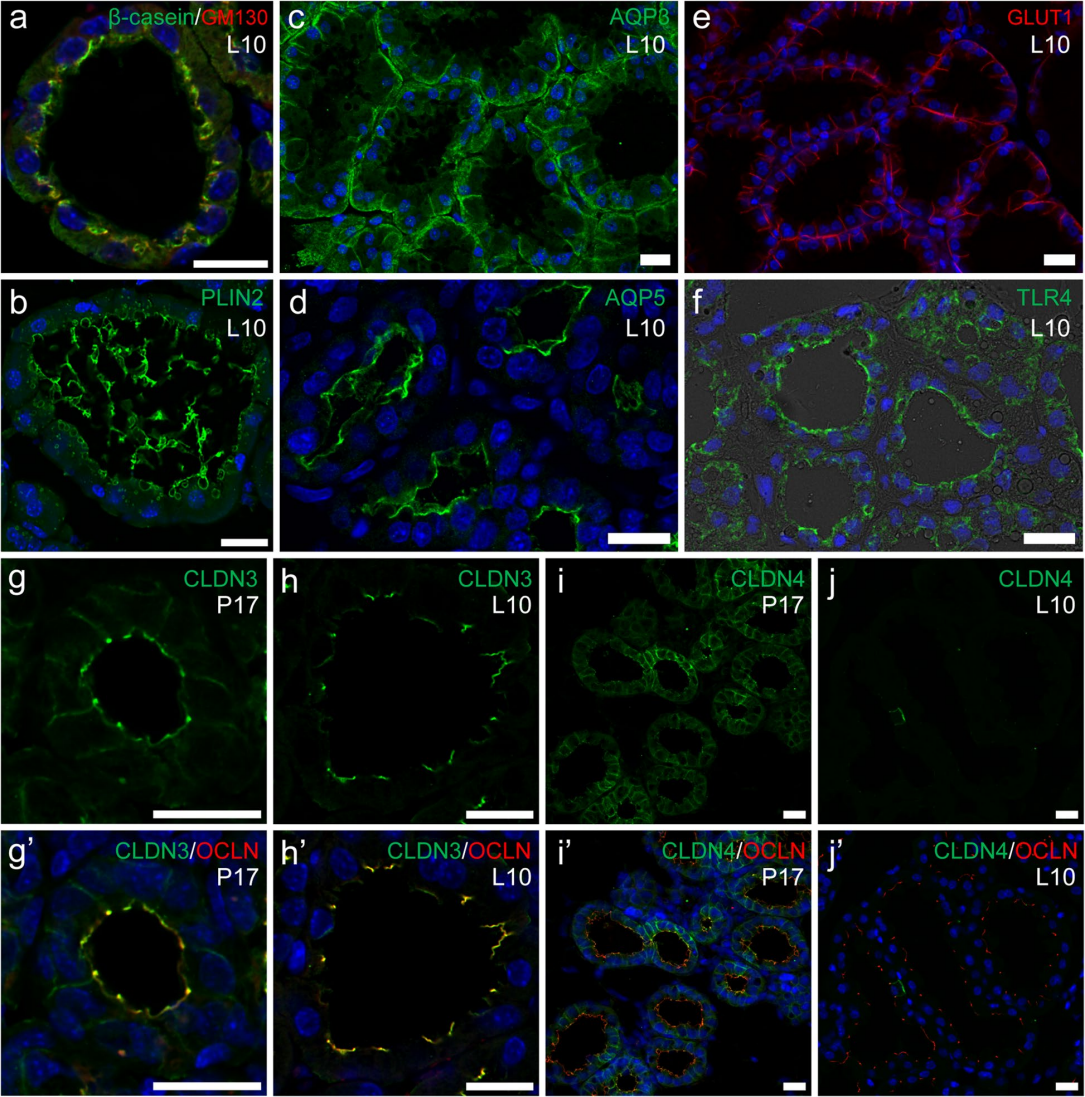
Milk production and TJs in MECs during lactation. **a**–**j’** Immunostaining images of β-casein (green; **a**) with GM130 (red; Golgi apparatus marker), PLIN2 (green; **b**; lipid droplet marker), AQP3 (green; **c**), AQP5 (green; **d**), GLUT1 (red; **e**), TLR4 (green; **f**), CDLN3 (green; **g**, **h, g’, h’**), and CLDN4 (green; **i**, **j**, **i**’, **j**’) with OCLN (red; **g’**–**j’**) in mammary alveoli of mouse mammary glands on pregnancy day 17 (P17; **g, g’, i, i’**) and lactation day 10 (L10; **a**–**f**, **h, h’, j, j’**) . Blue indicates the nuclei stained with DAPI. Scale bars are 20 μm

Alveolar MECs have complex and elaborate milk production pathways that are not found in other cell types [6–8]. Alveolar MECs absorb nutrients from the blood (Fig. 1). These include fatty acids, amino acids, and glucose. To efficiently absorb large amounts of nutrients, a wide variety of transporters and channels are expressed in the basolateral membrane of alveolar MECs [9–12]. After absorbing nutrients, alveolar luminal MECs synthesize casein and whey proteins using amino acids, and lactose using glucose and its metabolite (UDP-galactose). Caseins form micelles via calcium phosphate-mediated aggregation in the Golgi apparatus [13]. In addition, lactose is synthesized only in MECs via enzymatic reactions involving galactosyltransferase binding to α-lactalbumin, a protein specific to milk [14]. Synthesized milk proteins and lactose are packed in secretory vesicles and then released into the alveolar lumen by regulatory exocytosis (Fig. 2a) [15, 16]. Milk lipids are produced from fatty acids from the blood or by the de novo synthesis of medium-chain fatty acids in the endoplasmic reticulum of MECs [17]. The lipids are released into the cytoplasm as small droplets [18]. The lipid droplets gradually increase in size in the cytoplasm by repeated fusion and are directionally transferred toward the apical plasma membrane (Fig. 2b). The large lipid droplets are finally wrapped in the apical membranes and released into the alveolar lumen as milk fat globules by an apocrine mechanism. Thus, the milk production pathway proceeds from the basolateral membrane to the apical membrane of MECs.

A wide variety of factors is involved in the induction of milk production pathways in alveolar MECs. Prolactin and glucocorticoids are representative lactogenic hormones that activate the signal transducer and activator of transcription 5 (STAT5) and glucocorticoid receptor (GR) pathways, respectively. The prolactin/STAT5 pathway induces the expression of a set of genes involved in milk production, including milk proteins, lipids, and lactose [19–21]. GR is a nuclear receptor that is activated by binding of glucocorticoids. GR acts as a transcriptional coactivator of STAT5 to enhance the STAT5-dependent transcription of lactogenesis [22, 23]. Other endogenous factors, such estrogen, progesterone, serotonin, growth hormone, and inflammatory cytokines regulate milk production of MECs by activation with specific intracellular signaling pathways [24–29]. Exogenous factors, such lipopolysaccharide, lipoteichoic, isoflavones, menthol, and nicotine also regulate milk production of MECs [30–35].

### Tight Junction (TJ) Formation around Parturition

During lactation, alveolar MECs have less-permeable TJs at the most apical regions of the lateral membrane (Fig. 1) [36, 37]. The TJs of alveolar MECs are composed of claudin 3 (CLDN3) and occludin (OCLN) transmembrane proteins [38]. TJs function as a “barrier” to prevent leakage of milk and blood components via paracellular pathways between adjacent alveolar MECs (blood-milk barrier) [37]. In addition, TJs function as a “fence” because the transmembrane structures of TJs existing along the most apical regions of the basolateral membranes prevent the diffusion of phospholipids and membrane proteins in the cell membranes [39]. The cell membranes of alveolar MECs are compartmentalized on the basolateral and parietal sides along the TJ structure as the boundary [40]. In lactating mammary glands, alveolar MECs distinctly absorb nutrients from the basolateral membrane and secrete milk components through the apical membrane. The functional compartmentalization of cell membranes by TJs in alveolar MECs is also recognized by the different arrangements of membrane proteins containing channels, transporters, and receptors between the basolateral and apical membranes. For example, glucose transporter 1 (GLUT1) and aquaporin 3 (AQP3) are localized in the basolateral membranes of alveolar MECs that absorb nutrients, whereas receptors for sensing microbes and their toxins, such as Toll-like receptor 2 (TLR2) and TLR4, are expressed on the apical membranes (Fig. 2c–f) [41–43]. Thus, TJs are indispensable for the acquisition of cell polarity and appropriate physiological functions of alveolar MECs.

The TJ barrier in alveolar MECs is weak during pregnancy and prior to parturition [37]. However, the TJ barrier becomes less permeable, and less-permeable TJs are maintained throughout lactation. This functional change in TJs around parturition is concurrent with a decrease in the amount of CLDN4 and translocation of CLDN3 into TJs at the most apical regions of the basolateral membranes in alveolar MECs (Fig. 2g–j’) [38]. Alveolar TJs formed during lactation are mainly composed of CLDN3 and OCLN [44]. In addition to CLDN3, a lactation-specific increase in CLDN8 expression has been reported [45]. CLDNs form the strand structure of TJs [46]. Differences in the composition of CLDNs affect the properties of TJ barriers concerning the permeability of water and ions [47]. However, no such expressional change occurs for OCLN, although OCLN is employed as a general marker to indicate TJ regions during pregnancy and lactation. The change in CLDN composition is considered to reflect the change in the TJ barrier function from pregnancy to lactation.

Prolactin and glucocorticoids are representative lactogenic hormones that control the TJ barrier and the expression patterns of TJ proteins [37, 48]. Glucocorticoids increase the expression of both CLDN3 and CLDN4 in TJ regions [44]. In addition, glucocorticoids induce less-permeable TJs in cultured MECs and alveolar MECs of mice after adrenal gland removal [44, 49]. In contrast, prolactin decreases the expression of CDLN3 and CLDN4 and weakens the TJ barrier in cultured MECs [44]. In addition, administration of prolactin inhibitors (bromocriptine) to lactating mice increases CLDN4 expression in alveolar MECs. Interestingly, treatment with both prolactin and glucocorticoids induces high expression of CLDN3 and low expression of CLDN4 concurrently with an increase in epithelial barrier function in MECs in vitro, similar to the TJs of alveolar MECs in vivo [44]. Thus, prolactin and glucocorticoids induce lactation-specific TJs, in addition to inducing milk production in alveolar MECs around parturition.

### Dysfunction of MECs in Mastitis and after Weaning

Mastitis is an inflammation of mammary glands that is mainly caused by intramammary infections in cows [50]. Mastitis causes a decrease in milk quality and quantity in lactating mammary glands in cows [51]. At the cellular level, the expression levels of factors related to milk production are decreased in cultured mouse and bovine MECs in association with inactivation of STAT5 [30, 52]. In addition, the increase in CLDN4 expression and translocation of CLDN3 occurs concurrently with the weakness of the TJ barrier by injection of lipopolysaccharide, one of the endotoxins from *Escherichia coli* in mouse MECs [53]. The epithelial barrier of mammary glands is weak, and milk contains high concentrations of sodium and chloride in goats [37]. These reports indicate that mastitis causes weakness of the TJ barrier and decreased milk production. Furthermore, inflammatory signaling pathways, such as nuclear factor-kappa B (NF-κB) and STAT3 are activated, and the expression levels of proinflammatory cytokines are increased in bovine, goat, and mouse MECs [54–57]. Inflammatory cytokines also cause abnormalities in the milk production capacity of MECs and weaken the TJ barrier in bovine, rat, and mouse MECs [28, 58, 59].

Downregulation of milk production and TJ barrier function in alveolar MECs also occurs in weaning mammary glands in cows [60]. Mammary gland involution is divided into two phases. In mice, the first phase occurs within 48 h and second after 48 h [61]. In the first phase of involution, a gradual decline in the milk-producing ability occurs concurrently with an increase in CLDN4 in mice [38]. In addition, STAT5 and GR signaling are gradually inactivated, whereas STAT3 and NF-κB are activated in mice [62–64]. Thus, alveolar MECs in the first phase of involution and those in mastitis are similar concerning the activation of intracellular signaling pathways. When milking resumes, alveolar MECs recover from the negative effects of weaning during the first phase of involution. Such negative effects on alveolar MECs occur because of extended milking intervals (approximately 20 hours in cows), which would also be expected to act as a brake to suppress excess milk accumulation in the alveolar lumen in mice, dromedaries, cows, and ewes [65–68]. In contrast to the first phase of involution, the second phase of involution is irreversible in mice [69]. Massive loss of alveolar MECs is caused by anoikis after disruption of the surrounding basement membrane through activation of matrix metalloproteinases in mice [70]. Milk production pathways and TJ barriers in alveolar MECs no longer function during the second phase of involution.

As described above, alveolar MECs have a high milk production ability and a less-permeable TJ barrier during lactation. In contrast, the milk production ability of alveolar MECs in mastitis and early weaning is low and the TJ barrier is weak. Comparison of alveolar MECs between lactating, mastitic, and weaning mammary glands has revealed important intracellular signaling pathways that positively and negatively regulate milk production and the TJ barrier, such as the STAT5, GR, NF-κB, and STAT3 pathways. The activation state of these signaling pathways is an indicator of functional MECs in vitro*.*

## Overview of MEC Culture Models

### Functions Required for MECs in Culture Models

In lactating mammary glands, alveolar MECs function as secretory cells for milk production. To produce major milk components, alveolar MECs absorb abundant nutrients via the channels and transporters on the basolateral cell membrane, synthesize milk components intracellularly, and finally secrete them into the alveolar lumen via the apical membrane (Fig. 1). In addition, alveolar MECs block the leakage of blood and milk components via the paracellular pathway by less-permeable TJs. Furthermore, TJs compartmentalize the basolateral and apical membranes, which have distinctly different membrane proteins [39, 71]. These compositional differences in membrane proteins allow MECs to function differently on the parietal and basolateral sides and contribute to the acquisition of cell polarity [40, 72]. Therefore, induction of milk production and TJ formation is required for MECs in a culture model reflecting in vivo functional alveolar MECs. The TJ barrier can be evaluated by measuring transepithelial electrical resistance and the flux of fluorescein [44]. The transepithelial electrical resistance is a nearly instantaneous electrical assessment of ionic permeation through paracellular pathway, and the measurement of fluorescein flux allows the quantification of solute flux through the MEC layer over longer time periods [73–75]. In addition, we have previously reported that STAT5 and GR are activated in functional MECs in vitro, whereas the levels of NF-κB and STAT3 are low [30, 44, 57, 58, 76–78]. The functionality of MECs can be estimated by examining the activation levels of these signaling pathways.

### MECs for Preparation of Culture Models

**Fig. 3:**
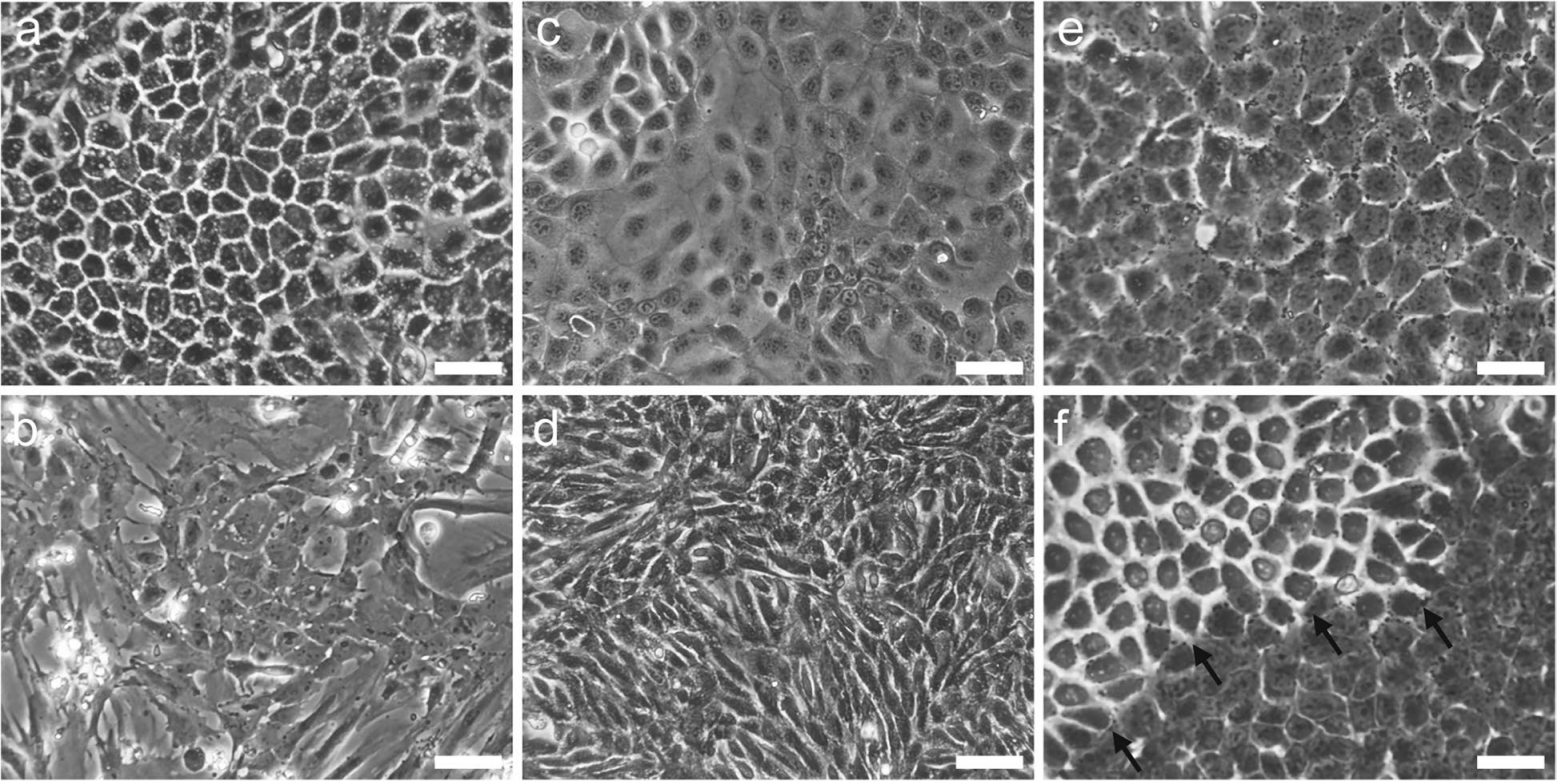
Phase-contrast microscopic images of MECs with or without passaging culture. **a** Phase-contrast microscopic images of primary MECs isolated from mouse (**a**) and bovine (**c**) mammary glands, mouse (**b**) and bovine (**d**) MECs at passage 2, and mouse mammary epithelial cell line HC11 cells from additional passage 0 (**e**) and 2 (**f**) after obtaining. Scale bars represent 50 μm. Arrows indicate a colony of HC11 cells, which underwent transformation during cultivation

The MECs used in culture models are broadly classified into primary cells and cell lines. Primary MECs are isolated from mammary glands of cows, sows, goats, mice, rats, rabbits, Guinea pigs, and humans [79–86]. They are commonly isolated by enzymatic treatment, mainly with collagenase. Y27632 dihydrochloride, a ROCK inhibitor, is used to avoid anoikis of MECs after isolation from the mammary glands since collagenase digestion triggers the loss of cell-matrix contact [87]. After enzymatic treatment, cells other than luminal MECs and myoepithelial cells, such as fibroblasts and adipose cells, are often removed using density gradient centrifugation or flow cytometry [44, 88–91]. The seeding density of MECs and number of culture passages have varied in different published studies. However, milk protein production ability of bovine MECs decreases as the number of passages increases [92, 93]. We have also confirmed that the induction of TJ formation in bovine MECs became more difficult as the number of cell passages increases (unpublished data). To prevent changes in properties due to passaging culture of MECs as shown in the phase contrast microscope image in Fig. 3a–d, the current practice in our laboratory is to seed primary mouse and bovine MECs isolated from both mouse and bovine mammary glands at a high density to reduce the cell proliferation frequency as much as possible. In addition, mammary epithelial fragments isolated from mammary glands can be cryopreserved as primary luminal MECs [88].

Cell lines established by transformation of primary MECs acquire unrestricted proliferative ability and uniform properties. Established MEC cell lines do not require cell isolation from the mammary glands before preparing the culture model. HC11 cell line is a prolactin-responsive cell clone derived from the COMMA-1D cells of mouse mammary epithelial cell line [94]. MAC-T cell line is established from primary bovine alveolar MECs [95]. Other cell lines from a wide range of animal species have also been reported. These include a human MCF-12A cell line [96] and goat MEC cell line [97]. These cell lines have contributed to research on breast cancer and mammary epithelial morphogenesis, in addition to milk production. However, cell lines sometimes show different properties from primary cultured MECs and other cell lines in vitro [98–102]. In addition, random transformation often occurs during passaging of cell lines by changing their properties. Figure 3e and f shows the colony of HC11 cells transformed during cultivation. Such transformation often occurs when the culture environment is unstable (e.g., irregular medium changes, medium pH fluctuations), as can occur when the experimenter is inexperienced with the culture technique. When using cell lines to prepare cell culture models, it is important to confirm that their characteristics are original and not transformed.

### Culture Substrates for Culture Models of MECs

Alveolar MECs require integrin-mediated cell adhesion to the basement membrane for lactogenic differentiation [103, 104]. The basement membrane is a thin sheet of extracellular matrix composed primarily of laminin and type IV collagen [105]. These basement membrane components are used for coating substrates on the dish or for the gel substrate of three-dimensional culture in MECs [106–108]. In particular, three-dimensional cultures of MECs in Matrigel form organoids with the alveolar-like luminal structure [109, 110]. When alveolar-like organoids are cultured in medium containing prolactin and glucocorticoids, milk components are secreted into the lumen [109, 111]. In this review, organoid refers to clusters of cells to form complex structures that partially resemble the in vivo organs and distinguishes from spheroids that are simple clusters of cells without forming complex structure. Depending on culture conditions, the clusters of MECs do not form complex structures as organoids but remain spheroids.

Fibrillar collagen is another representative substrate for MEC culture. Type I, III, and V collagens belong to the classical fibrillar collagen and constitute hybrid fibrils [112]. In mammary glands during pregnancy, MECs are surrounded by collagen fibrils that are composed mainly of type I collagen in mice [113]. Around the time of parturition, the fibrillar network of type I collagen becomes sparse and type III collagen is temporally upregulated in mammary glands. During lactation, the fibrillar network of type V collagen develops in connective tissues around the mammary alveoli. For MEC culture, collagen derived from rat tendons, bovine, or pig skins is used as a culture substrate [88, 114–116]. The main component of tendons and skin is type I collagen [117]. Collagen is used to coat dish surfaces and reconstitute a collagen gel. Interestingly, casein production is higher in bovine MECs seeded on floating collagen gels than on attached collagen gels [118]. In addition, excess deposition of type I collagen in mammary glands causes impaired mammary morphogenesis in cows [119]. However, it remains unclear whether and how much MECs contact with fibrillar collagens in vivo because the MECs are directly enclosed in basement membrane. In addition, the expression patterns of type I, III, and V collagens in conjunction with mammary remodeling during pregnancy, parturition, and involution remain unclear in most animal species containing cow. Furthermore, to the best of our knowledge, there are no examples of the use of fibrillar collagen other than type I collagen in culture.

### Two-Dimensional and Three-Dimensional Cultures of MECs

There are three major types of MEC culture models: two-dimensional culture in which MECs are seeded on a culture dish and form an epithelial sheet, insert culture in which MECs are seeded two-dimensionally on a semipermeable sheet and form an epithelial sheet, and three-dimensional culture in which MECs are embedded in a gel and form organoids. Two-dimensional culture on the culture dishes is the simplest and least expensive. The bottom surface of commercially available cell culture dishes is modified to support the growth and propagation of cells in culture, and MECs directly adhere to cell culture dishes in the absence of collagen or laminin. However, in the two-dimensional culture using culture dishes, nutrients for MECs and the milk components secreted from MECs are contained in the same medium. In contrast, commercially available cell culture inserts are useful for investigating the function of epithelial cells that are in contact with different liquid phases on the apical and basolateral sides. Materials used for the insert membranes are nitrocellulose or polyethylene terephthalate with pores 0.4 μm. MECs seeded on the cell culture insert are in contact with the medium from both the basolateral and apical sides [88]. MECs with well-developed TJs absorb nutrients contained in the lower medium through the pores of the insert membranes via channels and transporters in the basolateral membranes. In contrast, the soluble components in the upper medium are blocked from passing through the basolateral sides of MECs by TJs. In addition, cell culture inserts enable investigation of the influence of physiological components in milk (such as lipopolysaccharide) and blood (nutrients and growth factors) on the apical and basolateral membranes of MECs by adding them to upper and lower media, respectively, owing to the separation of the upper and lower media by the MEC layer and their TJs [57, 76]. Cell culture inserts also permit evaluations of the TJ barrier by measuring transepithelial resistance and leaked small molecules [44]. Furthermore, three-dimensional culture using Matrigel is an excellent method for reconstituting the alveolar-like luminal structure. The organoids initiate to secrete milk components into the lumen after treatment with prolactin and glucocorticoid. Sumbal et al. have also succeeded in reproducing the involution process of mammary epithelial structures using the three-dimensional cultures of organoids [120]. However, the running cost of the cell culture insert and Matrigel is much higher than that of the cell culture dish.

### Culture Temperature

Lactating mammary glands produce abundant metabolic heat during the process of milk component synthesis [121]. The body temperature of cows increases depending on the amount of synthesized milk components [122]. However, heat stress and excess metabolic heat production lead to a decline in milk production in dairy cows [123]. These reports indicate a close involvement between temperature and milk production in MECs. Previously, we cultured mouse MECs at 37, 39, and 41 °C in the presence of prolactin and dexamethasone [124]. The results showed that 39 °C treatment activated milk production and enhanced the formation of less-permeable TJs compared to 37 °C. In contrast, 41 °C treatment caused adverse effects on the TJ barrier and cell viability, although the milk production ability of MECs was temporarily up-regulated. Therefore, in our laboratory, mouse MECs for investigating milk production and TJ barrier are cultured at 39 °C. Since different animal species have different body temperatures, the optimal culture temperature for MECs may also vary among species.

### Culture Media Components that Induce MEC Function

For more than a half-century, prolactin, insulin, and glucocorticoid have been used as lactogenic hormones to induce milk production in MECs [125]. Prolactin and glucocorticoid activate the STAT5 and GR pathways, respectively, and induce lactation-specific TJ formation in addition to milk production in MECs [44]. Insulin is the principal metabolic regulator that promotes glycolysis in somatic cells and promotes cellular metabolism in association with mammary acinar development and milk production in MECs [126, 127]. In addition, epidermal growth factor (EGF), transferrin, estrogen, progesterone, serotonin, and growth hormone are involved in milk production in MECs [128–135]. Culture media containing additional nutrients, such as amino acids, vitamins, and fatty acids also regulate milk production in MECs in vitro [136–140]. In particular, effects of fatty acids on milk lipid production and size of milk fat droplets are different depending on the type of fatty acid [141, 142]. The abundant addition of these components to the medium may improve milk production by MECs. However, media components that induce high milk production are not always suitable for culture models to investigating unknown lactogenic components, such as plant galactagogues [143]. In addition, medium additives, which strongly activate certain intracellular signaling pathways, may obscure the regulatory mechanism of ingredients under study. Medium components need to be modified according to the purpose of the experiment.

## A Protocol for Establishment of MEC Culture with Functional TJ Barrier and Milk Production Capability Using Cell Culture Inserts

### Animals

Virgin ICR mice (9–13 weeks of age). All animal experiments must be approved by authorities and conducted in accordance with guidelines for the care and use of laboratory animals. In addition, personnel who actually perform animal experiments must have training prior to performing experimental manipulations.

### Materials and Tools

RPMI-1640 medium (Wako, Osaka, Japan, #189–02025), collagenase (Wako, #032–22,364, stock solution: 10 mg/mL in RPMI-1640), penicillin-streptomycin solution (10,000 U/mL penicillin, 10 mg/mL streptomycin, Wako, #168–23,191, the addition of antibiotics reduces the risk of contamination but not mandatory), trypsin (Wako, #207–19,982, stock solution: 20 mg/mL in phosphate buffered saline [PBS]), fetal bovine serum (Sigma-Aldrich, St. Louis, MO, USA), mouse EGF (Thermo Fisher Scientific, Waltham, MA, USA, #354001, stock solution: 20 μg/mL in PBS), insulin (Wako, stock solution: 10 mg/mL in 10 mM HCl), bovine pituitary extract (BPE; KURABO, Osaka, Japan, #KK-5102, prolactin can be used as a substitute for BPE), dexamethasone (Sigma-Aldrich, #D1756, stock solution: 10 mM in ethanol), cell culture dish (35 mm; Thermo Fisher Scientific, #353001), 24 well culture plate (Thermo Fisher Scientific, #353047), cell culture insert (Thermo Fisher Scientific, #353095, 0.4 μm, polyethylene terephthalate, 24-well format), forceps, scissors, scalpel, 15 mL and 50 mL centrifuge tubes. Stock solutions are stored at −80 °C. For short-term storage, −20 °C is fine.

### Medium Recipes

Growth medium is composed of 10 ng/mL EGF and 10 μg/mL insulin in RPMI-1640 medium with or without antibiotics. Differentiation medium is composed of 10 ng/mL EGF, 10 μg/mL insulin, 1 μM dexamethasone, and 0.1% BPE (or 50 ng/mL bovine prolactin) in RPMI-1640 medium. No need to add antibiotics in the medium if there is no risk of contamination. Antibiotics may influence cell growth and differentiation [144].

### Procedure for Isolation of Mammary Luminal Epithelial Fragments from One Mouse

**Fig. 4:**
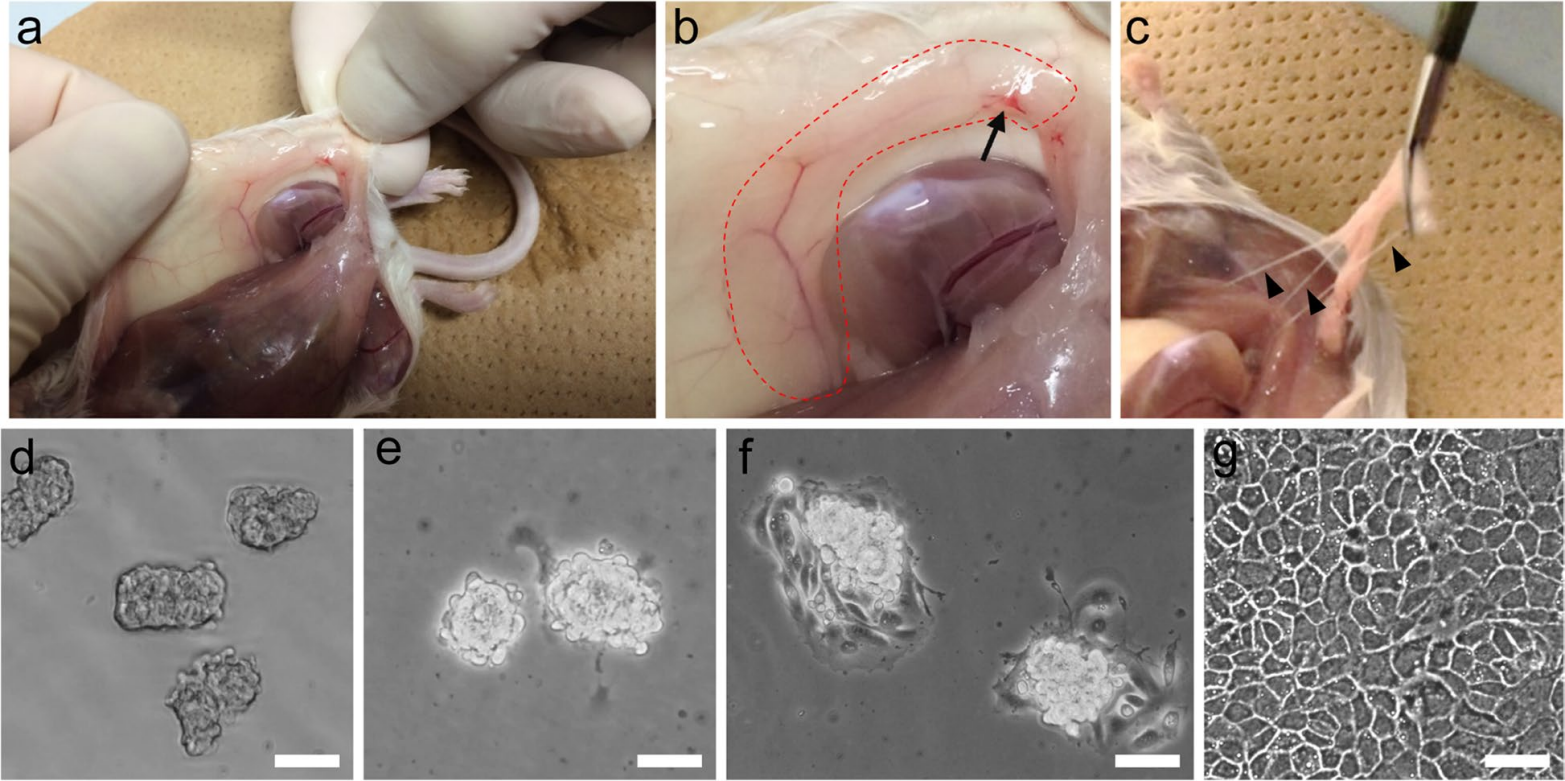
Isolation process of mammary epithelial fragments. **a** Photographs of skin underside of virgin ICR mouse. **b** A magnified image of **a**. The area enclosed by the dotted line shows a 4th mammary gland. An arrow indicates a lymph node in the mammary gland. **c** a 4th mammary gland detached with tweezers. Arrowheads indicate the membranous structure that connects the skin to the mammary gland and blood vessels attached to the mammary glands. **d**–**g** Phase-contrast microscopic images of mammary epithelial fragments isolated from 4th mammary glands of virgin ICR mice by collagenase treatment (**d**), epithelial fragments attached on the cell culture dishes 8 h (**e**) and 16 h (**f**) after seeding, and MECs 3 days after cultivation with the differentiation medium (**g**). Scale bars represent 50 μm

1. Sacrifice one mouse by cervical dislocation.
2. Remove the right and left 4th mammary glands using scissors and forceps (Fig. 4a–c).
3. Wash the mammary glands with PBS containing 100 U/mL penicillin and 100 μg/mL streptomycin on a clean bench.
4. Remove lymph nodes in mammary glands (Fig. 4b) and the interstitial membranous structure that connects the mammary glands to the skin and blood vessels attached to the mammary glands (Fig. 4c).
5. Mince mammary glands with a scalpel as quickly as possible (to approximately 0.5–1.0 mm^3^).
6. Digest chopped tissues in 20 mL of RPMI-1640 medium containing 7.5 mg/mL collagenase for 2 h at 37 °C with horizontal shaking at 70 rpm.
7. Mechanically disrupt undigested tissues by pipetting using a Pasteur pipette.
8. Centrifuge for 1 min at 600×*g* to collect cell fraction.
9. After discarding the supernatant, suspend cell fraction in 6 mL of RPMI-1640 medium containing 2 mg/mL trypsin and disperse by pipetting for 1 min at room temperature. Perform trypsin treatment weakly (by pipetting only a few times for a few seconds) if you wish to collect myoepithelial cells together with luminal MECs.
10. Centrifuge for 1 min at 600×*g* to collect cell fraction.
11. After discarding the supernatant, suspend cell fraction in 6 mL of RPMI-1640 medium containing 50–60% FBS.
12. Isolate luminal epithelial fragments by low-speed centrifugation for 5 min at 10×*g*; supernatant contains fibroblasts and myoepithelial cells.
13. After discarding the supernatant, wash cell fraction with 6 mL of RPMI-1640 medium and centrifuge for 1 min at 600×*g*.
14. Repeat steps 9–12.
15. Cell fraction obtained by the second low-speed centrifugation contains mammary luminal epithelial fragments for MEC culture (Fig. 4d).

### Procedure for Culture of MECs on Cell Culture Inserts

1. Suspend mammary luminal epithelial fragments from one mouse in 4 mL of growth medium. Adjust the volume of growth medium depending on the amount of pellet obtained (approximate concentrations are 5000 epithelial fragments/mL or 3.0 × 10^5^ cells/mL).
2. Add 0.8 mL of growth medium to each well of a 24-well culture plate.
3. Place the cell culture inserts in each well without air bubbles under the insert membrane.
4. Add 200 μL of suspension of mammary epithelial fragments to the interior of each cell culture insert.
5. Transfer plate to incubator and incubate at 37 °C in an atmosphere with 5% CO_2_ for 3–4 days. (Active migration of MECs from the fragments occurs within a day of seeding as shown in Fig. 4e and f.)
6. Change the growth medium in the upper and lower chambers and incubate for additional 2 days.
7. Change the growth medium to the differentiation medium both in the upper and lower chambers and incubate for 1.5 days at 37 °C.
8. Change the incubator temperature to 39 °C and incubate for additional 1–2 days.
9. Replace the differentiation medium in the upper chamber with RPMI-1640 medium. Medium in the upper and lower chambers is changed once a day after culturing at 39 °C.

### Expected Outcomes and Applications

**Fig. 5:**
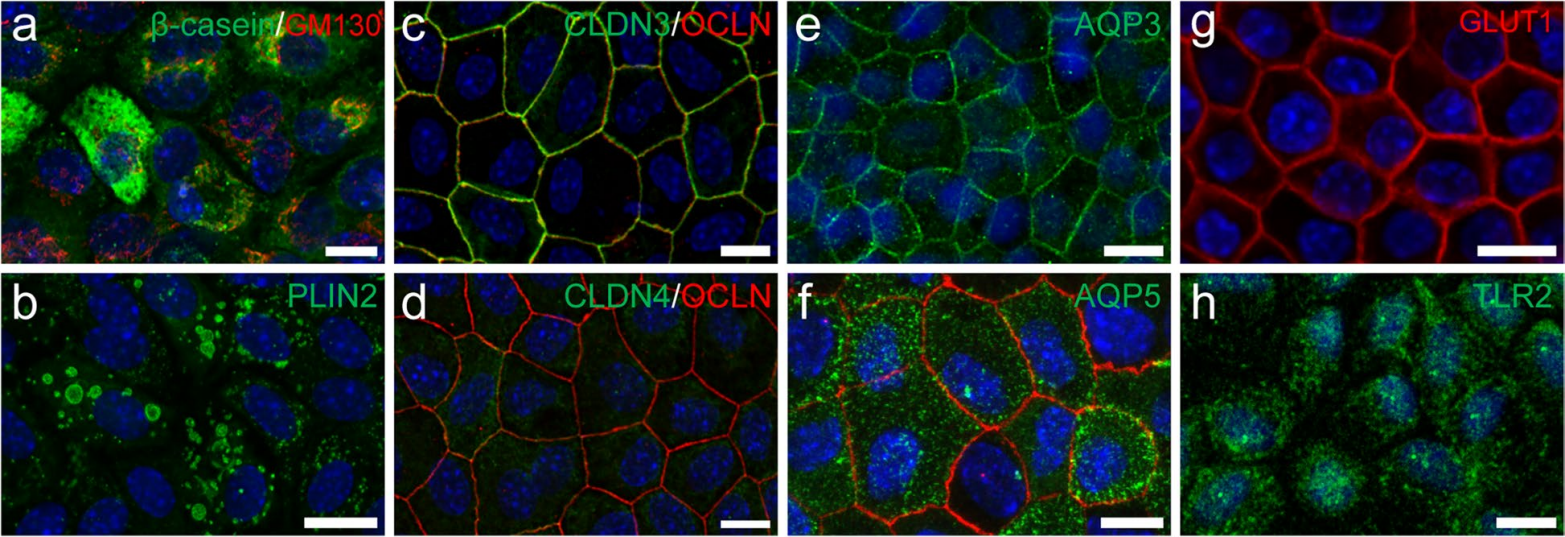
Immunostaining images of functional MECs in vitro. **a**–**h** Immunostaining images of β-casein (green; **a**) with GM130 (red; Golgi apparatus marker), PLIN2 (green; **b**; lipid droplet marker), CDLN3 (green; **c**), CLDN4 (green; **d)**, AQP3 (green; **e**), AQP5 (green; **f**), GLUT1 (red; **g**), TLR2 (green; **h**) with OCLN (red; **c, d, f**) in MECs on the cell culture inserts after 5 days of cultivation with the differentiation medium. Blue indicates the nuclei stained with DAPI. Scale bars are 10 μm

MECs on the cell culture insert after 1.5 days of culturing at 39 °C are available as the culture model for milk production and TJ barrier with specifically positioned membrane proteins (Figs. 4g and 5a–h). The formation of the TJ barrier can be noninvasively confirmed by measuring transepithelial electrical resistance using a Volt-Ohm Meter (for example, model ERS-2 from Merck). In addition, the induction of milk production can be confirmed noninvasively by examining the presence of milk components in the upper medium.

## Conclusions and Perspectives

Efforts to reduce animal experimentation are being actively pursued globally. In addition, research on milk production and TJ barrier in human MECs is required to solve breastfeeding problems. Therefore, functional MEC culture models reflecting alveolar MEC in vivo are expected to become increasingly necessary. MECs of the culture model discussed in this article produce milk and feature lactation-specific TJs and membrane proteins specifically positioned in the apical and basolateral membranes. In addition, three-dimensional cultures of the organoids of MECs reproduce the alveolar-like luminal structure in vitro. The organoids initiate to produce milk components and form TJs by treatment with lactogenic hormones.

However, many aspects of the current culture model of MECs do not reflect alveolar MECs in lactating mammary glands. For example, myoepithelial cells, fibroblasts, adipocytes, and immune cells are present around alveolar MECs in lactating mammary glands. In addition, alveolar MECs in lactating mammary glands are exposed to a wide variety of physical stimuli, including expansion pressure due to milk accumulation, fluctuations in udder temperature due to external temperature, metabolic heat of milk component synthesis, and fluid flow of milk. Therefore, it will be necessary to develop co-culture models with cells other than MECs and culture models combined with machines that can reproduce physical stimuli. Furthermore, various bioactive substances are secreted by organs other than the mammary gland and affect MECs in vivo. Therefore, it is also essential to finally confirm the reproducibility of findings obtained in culture models by in vivo experiments. Furthermore, most of experimental reagents such as recombinant proteins for cell culture are for mice and humans, whereas there are very few reagents for cows, pigs, dogs, cats, and goats. I am not sure to what extent the differences in animal species of these reagents affect the results of culture experiments, but it is true that they are an obstacle to experiments on cell culture from various animal sources. I strongly believe that the development of culture models and culture reagents will make it possible to determine the regulatory mechanism of milk production in MECs of various animal species in vitro.

## Data Availability

The datasets generated and/or analyzed during this study are available from the corresponding author upon reasonable request.
